# Hybrid Fractionation of Cowpea: Combining Dry and Wet Routes to Produce Versatile Protein Ingredients

**DOI:** 10.1111/1750-3841.71256

**Published:** 2026-07-13

**Authors:** Renata Fialho Teixeira, Clóvis Antônio Balbinot Filho, Jaíne Oliveira, Graziele Grossi Bovi Karatay, Cristiana Ambiel, Acácio Antonio Ferreira Zielinski

**Affiliations:** ^1^ Department of Chemical Engineering and Food Engineering Federal University of Santa Catarina Florianópolis Santa Catarina Brazil; ^2^ Science and Technology Department The Good Food Institute Brasil São Paulo Brazil

**Keywords:** alternative protein, antinutritional factors, black‐eyed pea, functionality, hybrid process

## Abstract

Cowpea (*Vigna unguiculata* L.) is an underutilized protein‐rich pulse with strong potential to diversify the alternative protein market, particularly through sustainable fractionation strategies. However, comparative insights into how dry and hybrid routes influence protein structure, functionality, and nutritional quality within the same processing stream remain limited. This study evaluated the nutritional, morphological, and techno‐functional properties of ingredients recovered via dry and hybrid (dry–wet) processes. Dry fractionation (milling and air classification) yielded a cowpea protein concentrate (CPC) with 47.5% yield and 54.6 g·100 g^−1^ protein (d.b.). CPC served as feedstock for wet extraction, producing a cowpea protein isolate (CPI) with a 45.0% process yield and 87.5 g·100 g^−1^ protein (d.b.). The hybrid route increased protein purity compared to dry processing while reducing solvent demand (water and pH‐adjusting solutions) per unit of protein relative to conventional wet extraction of whole flour. Both ingredients mainly consisted of glutamic acid, aspartic acid, leucine, and lysine. The amino acid score (AAS) met FAO/WHO requirements, except for sulfur‐containing amino acids, with in vitro protein digestibility‐corrected AAS of 66% (CPC) and 70% (CPI). Soaking (CPC‐S) markedly reduced tannins, phytates, and ash content through leaching. CPI exhibited higher solubility at alkaline pH and improved emulsifying, foaming, and water‐/oil‐holding capacities than CPC. Structural analyses indicated that the hybrid process produced a more homogeneous protein‐rich matrix with signs of aggregation and molecular rearrangement. In contrast, dry‐fractionated ingredients retained heterogeneous structures associated with protein–carbohydrate interactions. Overall, the hybrid route efficiently produced a high‐purity CPI, with improved functionality, and provided a promising route for developing high‐value cowpea‐derived ingredients for plant‐based food applications.

**Practical Applications:**

This study demonstrates an efficient strategy for converting cowpeas into high‐value protein ingredients using a combined dry and wet fractionation approach. By removing starch‐ and fiber‐rich components during the initial dry fractionation step, the process generates a protein‐enriched fraction that facilitates downstream extraction and can reduce the intensity of aqueous processing compared to conventional wet extraction from whole flour. The resulting ingredients exhibit enhanced nutritional quality and improved techno‐functional performance, including higher solubility, emulsifying capacity, and foaming properties. These attributes support their use across diverse food applications, including plant‐based beverages, meat analogs, bakery products, and nutritional formulations. Overall, this approach expands the use of cowpea as a versatile, sustainable, and competitive alternative protein source for contemporary food systems.

## 1 Introduction

The global demand for sustainable, nutritious protein sources continues to reshape the food industry and drive innovation in plant‐based ingredients. Market analyses and recent reviews indicate significant growth in the alternative protein sector (Malila et al. 2024; Medeiros et al. 2024), driven by increasing consumer awareness of environmental sustainability, health, and ethical practices in the production chain. Moreover, technological advances have improved the functional performance of plant‐based ingredients in food matrices (Aschemann‐Witzel et al. 2021). In this dynamic market, underutilized legume crops, such as cowpea (*Vigna unguiculata* L.), have garnered greater interest due to their nutrient‐rich profiles, combining high‐protein content, dietary fiber, and bioactive compounds, with resilience to climate stress and demonstrating strong techno‐functional potential following fractionation (Zolqadri and Li 2025). These qualities make cowpeas a promising feedstock for producing sustainable protein concentrates and texturized ingredients for meat analogs and other plant‐based functional foods and products.

To produce protein‐rich ingredients from pulses, two pathways are most commonly employed: dry fractionation and wet extraction. Dry fractionation methods (typically milling followed by air classification) separate protein and starch by size and density differences through an air current, avoiding solvents and minimizing thermal and mechanical stress. Dry fractionation enables the production of protein concentrates (40–65 g 100 g^−1^, d.b.) without the need for high temperatures, preserving nutrient and protein integrity and enabling a more rational use of water and energy (Pelgrom et al. 2013; Rivera et al. 2024; Vogelsang‐O'Dwyer, Petersen, et al. 2020). The only by‐product of dry fractionation is a starch‐rich flour that can be further valorized in food applications, such as bakery products, thickening systems, and gluten‐free formulations. However, dry fractionation methods can be less efficient at separating fractions with similar size and density distributions and are particularly limited in removing soluble or heat‐labile antinutritional factors (ANFs), which may negatively affect protein digestibility and nutritional quality (Teixeira et al. 2023).

On the other hand, fully wet methods (protein extraction followed by isoelectric precipitation) produce high‐purity protein isolates (>80 g 100 g^−1^), with greater stability and enhanced digestibility, but at the expense of large amounts of water and chemicals (Pulivarthi et al. 2023). A considerable energy input is also required to perform multiple steps of alkaline extraction, centrifugation, isoelectric precipitation, and neutralization (De Angelis et al. 2024). In addition to wastewater and residue (nonprotein fraction) generation, salt accumulation in the final product resulting from sequential pH adjustments is problematic because of the high sodium content in the isolates, as observed for soy protein isolates (Peng et al. 2023).

Therefore, the so‐called hybrid process (dry fractionation followed by wet extraction) for producing protein isolates has emerged as a well‐optimized solution for the industry to achieve high‐protein recovery and superior nutritional and functional quality, as demonstrated in comparative assessments (Avila Ruiz et al. 2016; Berghout et al. 2015; De Angelis et al. 2024; Dumoulin et al. 2021). In these studies, the hybrid approach showed reduced resource demands for water, energy, and by‐product generation compared to fully wet purification for obtaining plant protein ingredients. A techno‐economic assessment of hybrid fractionation revealed moderate trade‐offs relative to dry fractionation and significant improvements over the wet route for achieving protein with purity equivalent to that of dry fractionation (Allotey et al. 2026). Still, a head‐to‐head comparison between protein concentrates and isolates produced from the same processing stream is a knowledge gap that needs to be addressed as processors seek to select the best fractionation route in a fit‐for‐purpose approach.

Notwithstanding, the nutritional quality of plant‐based proteins is primarily determined by the balance among their amino acid composition, digestibility, and the presence of ANFs. Protein digestibility is a key determinant of amino acid bioavailability and nitrogen utilization and has been commonly assessed using in vitro protein digestibility (IVPD) and derived metrics, such as the IVPD‐corrected amino acid score (IV‐PDCAAS). These metrics are critical not only for benchmarking plant‐based protein ingredients against traditional sources (Calvez et al. 2024; Nosworthy et al. 2017; Nosworthy and House 2017) but also for defining their nutritional values and guiding their application in food systems.

Cowpea protein is recognized as rich in lysine and branched‐chain amino acids (BCAAs, namely, leucine, isoleucine, and valine) but relatively low in sulfur amino acids (SAAs), such as methionine and cysteine (Abebe and Alemayehu 2022; Raizada et al. 2023; Shevkani et al. 2025; Vasconcelos et al. 2010). However, the presence of ANFs, such as protease inhibitors and nonprotein ANFs (phytates, tannins, and saponins), can negatively affect these metrics by forming indigestible complexes or inhibiting digestive enzymes (Cruz et al. 2004; Teixeira et al. 2023). Therefore, the impact of posttreatments, such as soaking dry‐fractionated protein concentrate to reduce the ANF load, warrants further evaluation, as this practice is well established for raw grains but remains less explored at an ingredient level.

This study aimed to analyze the compositional, nutritional, structural, and techno‐functional features of cowpea‐derived ingredients, namely, a protein concentrate produced via dry fractionation and a protein isolate produced by a hybrid (dry/wet) process. By exploring the dry–wet synergy, this work aims to clarify how fractionation pathways and soaking treatment influence the nutritional, structural, and techno‐functional properties of cowpea‐derived ingredients. This investigation also seeks to highlight the potential of a hybrid approach to produce alternative proteins with improved functionality and reduced environmental impact, aligning with the food industry's growing preference for more sustainable processes.

## 2 Materials and Methods

### 2.1 Materials

Dehulled *V. unguiculata* beans (density of 0.81 g cm^−3^ and humidity of 10.8%) were donated by LC Sementes (Sorriso, MT, Brazil) and shipped to Neuman & Esser (NEA) Test Center in Belo Horizonte, MG, Brazil, where they were milled and dry‐fractionated to obtain a starch‐rich cowpea flour (CF) and a protein‐rich concentrate (CPC).

Chemicals and reagents utilized for characterization analysis were of analytical grade and provided by external suppliers. The listing of names, uses, and manufacturers’ locations is summarized in the Supporting Information.

### 2.2 Cowpea Protein Fractions

#### 2.2.1 CPC Obtained by Dry Fractionation

Air classification was employed to fractionate cowpeas into a protein‐rich fraction (CPC) and a starch‐rich fraction (CF). Cowpea seeds (800 kg) were milled using an impact classifier mill (Neuman & Esser, ICM 630, Brazil) equipped with a rotative cylindrical classifier to obtain a fine flour (*d*_97_ = 33 µm). Milling was conducted under dry conditions to achieve a particle size distribution suitable for subsequent air classification. The ground flour was subsequently air‐classified using a guide ring classifier (Neuman & Esser, GRC 430, Brazil). Operational parameters were optimized to enhance the protein‐starch separation. These operating conditions enabled the separation of two distinct fractions: a fine, light fraction enriched in protein (CPC) and a coarse, heavier fraction predominantly composed of starch granules. The CPC fraction was selected for further processing and characterization.

**FIGURE 1:**
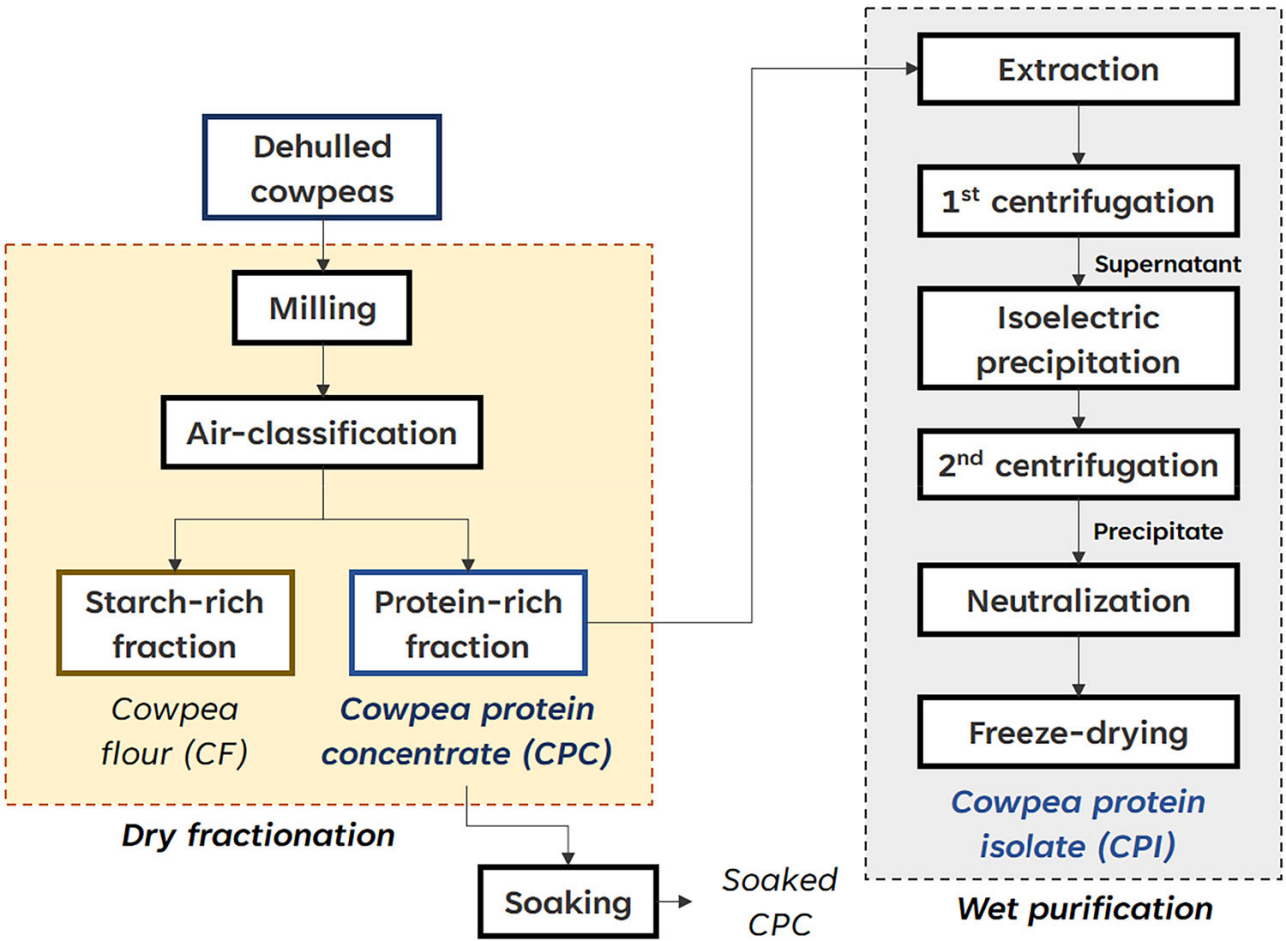
Flowchart outlining the main steps for obtaining the ingredients of cowpea beans. CF, cowpea flour; CPC, cowpea protein concentrate; CPI, cowpea protein isolate.

Additionally, the CPC underwent a soaking treatment to evaluate its effectiveness in reducing ANFs. CPC was suspended in deionized water at a 1:10 w/v ratio and maintained at 25°C for 16 h under static conditions (no stirring). This soaking approach is consistent with commonly applied treatments for legumes aimed at reducing ANFs through diffusion and solubilization mechanisms (Ibrahim et al. 2002; Khattab and Arntfield 2009). The soaked suspension was centrifuged at 2480 × *g* for 15 min (SOLAB, SL‐707, Brazil) to separate the supernatant, which was discarded. The resulting pellet was collected and freeze‐dried (Liotop L101, Brazil) to obtain the treated ingredient (CPC‐S). The flowchart for obtaining cowpea‐derived ingredients via dry fractionation and hybrid methods is schematized in Figure 1.

#### 2.2.2 Cowpea Protein Isolate (CPI) by Wet Process

The CPI was obtained from untreated CPC through conventional heating and stirring alkaline extraction followed by isoelectric precipitation (AE‐IEP), as previously described by Teixeira et al. (2024). Aqueous extraction was performed at a solid‐to‐liquid ratio of 1:10 (w/v) using alkaline water (pH 9.0). The suspension was stirred at 1035 rpm and 50°C for 1 h (IKA RW 20 digital, Brazil) and then centrifuged to recover the protein‐rich supernatant. Protein precipitation was induced at pH 4.5, corresponding to the isoelectric point (IEP) of cowpea proteins (determined from *ζ*‐potential measurements as a function of pH, Figure S1). The slurry was collected by centrifugation, resuspended in deionized water at a 1:20 (w/v) ratio, and homogenized in a tube shaker (Fisatom, 772, Brazil). The suspension was then neutralized to pH 7.0 (using 0.1 mol L^−1^ NaOH and HCl), without a subsequent washing step, and the final product was freeze‐dried to obtain CPI in a powdered form.

### 2.3 Proximal Composition

The proximate composition of CF, CPC (unsoaked), CPC‐S, and CPI was determined according to AOAC (2005) official methods: moisture (925.09); ash (923.03); total lipids (920.39); nitrogen content by standard Kjeldahl method (954.01) using a conversion factor of 6.25; and total dietary fiber (TDF) (985.29). Total carbohydrates were calculated by difference. Starch content was determined only in the CF fraction using the enzymatic method as described by Demiate et al. (2001).

### 2.4 Mineral and Metal Composition

The elemental mineral composition of CPC and CPI (calcium, copper, iron, and zinc), as well as heavy metal concentrations (cadmium, lead, and arsenic), were quantified by the analytical technique of multielement determination by Inductively Coupled Plasma Optical Emission Spectrometry (ICP‐OES: MY14020006, Agilent 700 Series, USA) according to EPA method 6010D, following acid digestion (Method 3050B) (US EPA 1996, 2014). Sodium chloride (NaCl) was reported as total salt content (expressed as NaCl equivalent) on the basis of analytical estimation and is therefore presented separately from the elemental mineral profile. All sampling and handling procedures followed laboratory‐scale adaptations of the Standard Methods for the Examination of Water and Wastewater (SMWW) protocols (APHA et al. 2023).

### 2.5 Structural Properties

#### 2.5.1 Scanning Electron Microscopy (SEM)

A SEM (Tescan Vega, Czechia) was used to examine the microscopic structure of cowpea ingredients (CF, CPC, CPC‐S, and CPI). A thin layer of sample was directly applied to double‐sided adhesive carbon tape on an aluminum stub and was coated with gold. Samples were examined at an accelerating voltage of 10 kV (1000–7000× magnification).

#### 2.5.2 Electrophoresis Analysis

The molecular weight (MW) distributions of the CPC, CPC‐S, and CPI were visualized by sodium dodecyl sulfate–polyacrylamide gel electrophoresis (SDS–PAGE) under reducing conditions. Protein ingredients were diluted 1:1 (v/v) with 2× Laemmli sample buffer (Bio‐Rad, Hercules, CA, USA), containing 5% β‐mercaptoethanol as the reducing agent, then heated at 95°C for 7 min. Proteins were separated in the 10–250 kDa range using a 4%–15% acrylamide Criterion TGX Precast Midi Protein Gel (Bio‐Rad, Hercules, CA, USA). The Precision Plus Protein Kaleidoscope Standards (Bio‐Rad, Hercules, CA, USA) were used as MW markers. Approximately 35 µg of protein was loaded into each well, and separation was performed at 200 V. Proteins were stained using Bio‐Safe Coomassie Blue Premixed Staining Solution and imaged using a Bio‐Rad Gel Doc EZ Imager (Bio‐Rad, Hercules, CA, USA).

#### 2.5.3 Fourier‐Transform Infrared Spectroscopy (FTIR) and Protein Secondary Structure

Infrared spectroscopy was performed using a Shimadzu instrument (IR Prestige‐21 FTIR spectrophotometer). Protein samples (∼1 mg) were mixed with KBr (100 mg), ground, and pressed into pellets. Spectra were recorded in absorbance mode from 4000 to 400 cm^−1^ at a resolution of 4 cm^−1^ with 32 scans. The Amide I region (1600–1700 cm^−1^) was analyzed to estimate protein secondary structure. Spectral processing included baseline correction and normalization, followed by second derivative analysis. Deconvolution of overlapping bands was performed using a constrained Gaussian fitting approach with a fixed number of components, on the basis of literature‐reported band assignments for protein secondary structures. Peak areas were obtained by integration of the fitted bands using OriginPro software (OriginLab, Northampton, USA), and results were expressed as the relative contribution (%) of each structural element.

#### 2.5.4 Differential Scanning Calorimetry (DSC)

The thermal properties of the cowpea protein ingredients were evaluated using a differential scanning calorimeter (Jade‐DSC, PerkinElmer, USA). Each sample of 5 mg (dry powder) was accurately weighed into aluminum pans and hermetically sealed. Samples were heated from 25°C to 200°C at 10°C min^−1^ under a nitrogen atmosphere (N_2_ flow rate: 25 mL min^−1^), as described by Wang et al. (2021), with minor modifications. Thermal transitions were characterized by the apparent peak temperature (*T*p), defined as the temperature at the maximum of the endothermic event, and the enthalpy change (Δ*H*), calculated by integrating the area under the peak after linear baseline correction using OriginPro (OriginLab, Northampton, USA).

### 2.6 Nutritional Profile

#### 2.6.1 In Vitro Protein Digestibility

IVPD was determined according to Hsu et al. (1977) with modifications (Sá et al. 2021; Tinus et al. 2012). Protein suspensions (6.25 mg mL^−1^) were adjusted to pH 8.0 under stirring at 37°C. An enzyme solution comprising (1.3 mg mL^−1^ peptidase, 1.6 mg mL^−1^ trypsin, and 3.1 mg mL^−1^ α‐chymotrypsin) kept on ice was also adjusted to pH 8.0 and added to the protein suspensions at a 1:10 (v/v) ratio at constant stirring (37°C). The resulting enzyme‐to‐substrate (E:S) ratios were 0.021, 0.026, and 0.050 mg of peptidase, trypsin, and α‐chymotrypsin per mg of protein, respectively. The rapid pH decrease, caused by the release of carboxyl groups during proteolysis, was monitored and measured after 10 min using a pH meter (Hanna HI5221, Romania). The percent IVPD was estimated using Equation (1), where ΔpH_10 min_ is the pH change at 10 min:

(1) $$\mathrm{IVPD}\;\left(\%\right)=65.66+18.10\times \Delta pH_{10\;\min}$$

#### 2.6.2 Amino Acid Profile

The amino acid (AA) profile of cowpea protein ingredients was determined using the Pico‐Tag method (Hagen et al. 1989; White et al. 1986). Initially, samples underwent acid hydrolysis with HCl (6 mol L^−1^) at 110°C for 22 h and were derivatized with phenylisothiocyanate (PITC). As performic acid oxidation was not performed before hydrolysis, the SAAs were not stabilized, potentially leading to partial degradation. However, all samples were processed under identical conditions to ensure a valid comparative interpretation. Tryptophan (Trp) was determined separately following alkaline hydrolysis (Lucas and Sotelo 1980). Chromatographic separation was performed in a high‐performance liquid chromatography (HPLC) system (Alliance e2695, Waters, USA) equipped with a fluorescence detector (2475, Waters, USA) using a reverse‐phase column (InfinityLab Poroshell 120 EC‐C18, Agilent Technologies). The mobile phase consisted of a gradient mixture of (A) aqueous buffer containing sodium acetate and (B) acetonitrile/water solution (60:40 v/v). The gradient program was as follows: 0% B (0 min), 2% B (0.5 min), 7% B (15 min), 9% B (19 min), 12% B (22 min), and 30% B (33 min), followed by column washing and reconditioning. Fluorescence detection was performed with excitation at 250 nm and emission at 395 nm. Then, the amino acids were identified using authentic standards and quantified by internal (α‐aminobutyric acid) and external calibration. Results were expressed as g 100 g^−1^ of sample.

The amino acid score (AAS) was calculated according to Sá et al. (2023, 2021) using Equation (2). Amino acid values were normalized to protein content and expressed on a protein basis. The AAS corresponds to the ratio between the content of a given essential amino acid (EAA) in 1 g of test protein and the corresponding requirement standard content (FAO/WHO 1991). The lowest AAS value indicates the most limiting amino acid (Nosworthy et al. 2018). The IV‐PDCAAS was calculated by multiplying AAS by IVPD (Nosworthy et al. 2018), using the FAO/WHO (1991) reference pattern for preschool‐aged children (2–5 years):

(2) $$\begin{array}{ccc} \mathrm{AAS}\;(\%) & = & \frac{\mathrm{mg}\;\mathrm{of}\;\mathrm{AA}\;\mathrm{in}\;1\;g\;\mathrm{of}\;\mathrm{test}\;\mathrm{protein}}{\mathrm{mg}\;\mathrm{of}\;\mathrm{AA}\;\mathrm{required}\;\mathrm{per}\;g\;\mathrm{of}\;\mathrm{reference}\;\mathrm{protein}(\mathrm{FAO}/\mathrm{WHO})}\\ & & \times \;100 \end{array}$$

#### 2.6.3 Antinutritional Factors

The ANFs of cowpea protein ingredients were quantified in raw CPC, CPC‐S, and CPI to evaluate the impact of processing.

##### 2.6.3.1 Phytic Acid

Phytic acid content in the samples was determined using the H‐L method (Haug and Lantzsch 1983), with adaptations from Raboy et al. (2017). Briefly, 0.25 g of the sample was extracted with 5 mL of HCl (0.2 N) in a boiling water bath for 1 h. Aliquots (250 µL) of the extracts were transferred to test tubes containing 2.25 mL HCl (0.2 N) and 5 mL of ferric reagent (ammonium iron(III) sulfate dodecahydrate). The mixture was boiled for 30 min, cooled to room temperature, and centrifuged (2841 × *g*, 10 min). Supernatant aliquots (100 µL) were reacted with 150 µL of the H‐L reagent (composed of 400 mg 2,2′‐bipyridine, 400 µL thioglycolic acid, and 40 mL deionized water). Then, the absorbance was measured at 519 nm using a microplate reader (Agilent Biotek Epoch, USA). Quantification was performed using a standard curve (*y* = −2.2123*x* + 0.7496; *R* ^2^ > 0.9995) of sodium phytate hydrate solution, with results expressed as milligrams of phytic acid equivalents per gram of sample (mg PAE g^−1^).

##### 2.6.3.2 Total Tannins

The total tannin content was determined by colorimetric analysis using the Folin–Ciocalteu reagent, following the methodology described by Ranganna (1986), with modifications proposed by Mumtaz Hamdani and Ahmed Wani (2017) and Nidhina and Muthukumar (2015). Briefly, 0.5 g of the sample was extracted with 80 mL of deionized water in a boiling water bath for 30 min, followed by centrifugation (2000 × *g*, 10 min). Supernatant aliquots (100 µL) were mixed with 500 µL of 50% Folin–Ciocalteu reagent and 1 mL of saturated sodium carbonate solution. The mixtures were incubated for 30 min at room temperature, and the absorbance was measured at 760 nm. Quantification was performed using a standard curve of tannic acid (*R* ^2^ > 0.993), and results were expressed as milligrams of tannic acid equivalents per gram of sample (mg TAE g^−1^).

##### 2.6.3.3 Trypsin Inhibitor Activity (TIA)

The TIA was determined according to the methodology described by Kakade et al. (1974), with slight modifications by Nidhina and Muthukumar (2015). The chromogenic substrate Nα‐benzoyl‐dl‐arginine‐*p*‐nitroanilide hydrochloride (BAPNA) was used for the assay. For extraction, 0.5 g of the sample was suspended in 25 mL of NaOH (0.01 N) and stirred for 3 h at room temperature. The suspension was centrifuged at 3000 × *g* for 10 min, and 1 mL of the supernatant was transferred to test tubes containing 1 mL of deionized water; blanks were prepared with deionized water only. Subsequently, 2 mL of standard trypsin solution (0.02 mg mL^−1^ in 0.001 mol L^−1^ HCl) and 5 mL of BAPNA solution (0.4 mg mL^−1^ Tris buffer 0.05 mol L^−1^, pH 8.2 containing CaCl_2_) were added to the tubes. The reaction mixtures were incubated at 37°C for 10 min, after which 1 mL of acetic acid (30%, v/v) was added to stop the reaction. Absorbance was measured at 410 nm, and TIA was expressed as the increase in absorbance of 0.01 units per 10 mL of reaction mixture, reported as trypsin inhibitor units per milligram of sample (TIU mg^−1^).

#### 2.6.4 Techno‐Functional Properties of Cowpea Protein Fractions

##### 2.6.4.1 Protein Solubility

Protein solubility was evaluated as a function of pH, as described by Ibrahim et al. (2021) and Jiang et al. (2017), with minor modifications. Samples (0.1 g) were dispersed in 10 mL of deionized water and homogenized using a vortex. The pH was adjusted to 3.0–9.0 using NaOH or HCl (0.1 mol L^−1^). The dispersions were shaken in a Dubnoff bath (304‐D Ethink, Brazil) for 30 min at room temperature. Subsequently, 2 mL of each suspension was centrifuged (12,879 × *g*, 15 min). The soluble protein content in the supernatant was quantified using a Bio‐Rad Protein Assay kit, on the basis of the Bradford method (Bradford 1976). For the assay, 20 µL of sample was mixed with 1 mL of dye reagent and incubated at room temperature for 5 min. Absorbance was measured at 595 nm, and protein concentration was calculated from a standard curve (*y* = 0.2842*x* + 0.0497, *R* ^2^ > 0.9969) prepared with bovine serum albumin (BSA, 2 mg mL^−1^). Protein solubility (%) was expressed as the percentage ratio of soluble protein content in the supernatant to the total protein initially present in the dispersion.

##### 2.6.4.2 Emulsifying Activity Index (EAI) and Emulsion Stability Index (ESI)

EAI and ESI were determined according to the method of Pearce and Kinsella (1978) as described by Gundogan and Can Karaca (2020), with slight modifications. Aqueous solutions of CPC and CPI (5 g L^−1^) were prepared, and the pH was adjusted to 7.0 with NaOH or HCl (0.1 mol L^−1^). Subsequently, 20 mL of commercial sunflower oil was added to each solution, and the emulsification was performed using a high‐speed homogenizer (Ultra‐Turrax T18 Digital, IKA, probe S 25 KV‐18 G) operating at 9500 rpm for 1 min at room temperature. Immediately after homogenization, a 50 µL aliquot of the emulsion was diluted in 5 mL of 0.1% (w/v) SDS solution and vortexed for 30 s. Absorbance was measured at 500 nm immediately (*A*_0_) and after 10 min (*A*_10_), and EAI and ESI were calculated using the following equations:

(3) $$\mathrm{EAI}\;\left(\frac{m^{2}}{g}\right)=\frac{2\times 2.303\times A_{0}\times N}{c\times \phi \times 10,000}$$

(4) $$\mathrm{ESI}\;\left(\min\right)=\frac{A_{0}}{\Delta_{A}}\times t$$

where *A*_0_ is the absorbance immediately after homogenization, *N* is the dilution factor, *c* is the protein concentration in the dispersion (g mL^−1^), ϕ is the oil volume fraction of emulsion (mL mL^−1^), Δ*_A_* is the difference between *A*_0_ and *A*_10 min_, and *t* is the time interval (10 min).

##### 2.6.4.3 Foaming Capacity (FC) and Foam Stability (FS)

FC and FS were determined according to the methodology described by Gundogan and Can Karaca (2020). Dispersions of CPC and CPI were prepared in deionized water (1% w/v) and adjusted to pH 7.0 with NaOH or HCl (0.1 mol L^−1^). Aliquots (10 mL) of each sample were transferred into tubes and homogenized using a high‐speed disperser (Ultra‐Turrax, IKA, model T18) at 13,500 rpm for 2 min at room temperature. Foam volume was recorded immediately after homogenization (time zero) and at 10, 30, and 60 min. FC was calculated as the percentage increase in the foam volume as a result of whipping (Equation 5), and FS was expressed as the percentage of foam volume retained over time (Equation 6):

(5) $$\mathrm{FC}\;\left(\%\right)=\frac{V_{2}-V_{1}}{V_{1}}\times 100$$

(6) $$\mathrm{FS}\;\left(\%\right)=\frac{V_{3}}{V_{2}}\times 100$$

where *V*_1_ is the suspension volume before whipping (10 mL), *V*_2_ is the foam volume immediately after homogenization (time zero), and *V*_3_ is the foam volume measured at each time interval (10, 30, or 60 min).

##### 2.6.4.4 Oil Holding Capacity (OHC) and Water Holding Capacity (WHC)

The WHC and OHC were determined according to the methodology described by Gundogan and Can Karaca (2020). CPC and CPI samples (0.1 g) were weighed (*M*_0_) into pre‐weighed microcentrifuge tubes (*M*_t_) and then mixed with 1.0 mL of deionized water (for WHC) or sunflower oil (for OHC). The mixtures were vortexed for 1 min and allowed to stand at room temperature for 30 min. Subsequently, the microtubes were centrifuged at 15,000 × *g* for 20 min, and the supernatant was carefully removed. The final mass of the microtubes containing the pellet (*M*_1_) was recorded. WHC and OHC were calculated using Equation (7) and expressed as grams of water or oil retained per gram of sample (ingredient basis), reflecting the functional behavior of the ingredients in their intact compositional matrix:

(7) $$\mathrm{WHC}\;\mathrm{or}\;\mathrm{OHC}=\frac{M_{1}-M_{t}-M_{0}}{M_{0}}$$

where *M*_0_ is the sample mass, *M*_t_ is the mass of the empty microtube, and *M*_1_ is the mass of the microtube after centrifugation.

##### 2.6.4.5 Gelling Ability

The gelling abilities of CPC and CPI were assessed qualitatively using the protocol of Sathe and Salunkhe (1981), with modifications as described by Ghribi et al. (2015). A series of protein dispersions was prepared at increasing concentrations, ranging from 20 to 200 mg mL^−1^ for CPC, and from 5 to 200 mg mL^−1^ for CPI. The samples were transferred to capped test tubes, vortexed for 1 min, and then heated in a water bath at up to 100°C for 1 h. Afterward, the tubes were rapidly cooled in an ice bath and stored at 4°C for 2 h. Gel formation was evaluated by inverting the tubes upside down and observing the flow behavior. The samples were categorized as follows: (−) the absence of gel or flow; (±) a weak gel, identified by a viscous flow; and (+) gel formation in the absence of flow. The minimum concentration at which the sample did not flow upon inversion was recorded as the least gelling concentration (LGC).

#### 2.6.5 Statistical Analysis

All assays were performed in triplicate, and results were presented as means ± standard deviation. One‐way analysis of variance (ANOVA) was applied to detect significant differences (*p* < 0.05). Fisher's least significant difference (LSD) test (*α* = 0.05) was applied for mean comparisons. Statistical analyses were performed using Statistica v. 13.5 (TIBCO Software Inc., Palo Alto, CA, USA).

## 3 Results and Discussion

### 3.1 Proximate Composition of Cowpea Ingredients

Cropping systems, environmental conditions, genotype, and processing techniques all influence the macronutrient composition of cowpea‐derived ingredients. Table 1 presents the proximate composition (d.b.) of the dry fractionation products: the starch‐rich coarse fraction, namely, CF, the protein‐enriched fine fraction (CPC) before and after soaking treatment (CPC‐S), and the CPI. Regarding dry fractionation, a nutrient redistribution occurred between CF and CPC, with significant differences (*p* < 0.05) for all constituents when taking into account the composition reported in the Brazilian Food Composition Table (NEPA 2011) for fresh cowpeas (20.2% protein, 2.4% lipids, 3.5% ashes, 23.6% TDF, and 70.1% total carbohydrates). The protein was concentrated in the fine portion (CPC, 54.6% d.b.), and CF was predominantly composed of carbohydrates (68.8% starch). According to Skylas et al. (2023), the protein content in the finer portion after dry fractionation varies from 40 to 65 g 100 g^−1^, depending on the pulse type, and the starch content in the coarser fraction ranges from 61 to 66.3 g 100 g^−1^.

**TABLE 1:** Proximate composition of cowpea ingredients.

Ingredient	Moisture^1^	Protein	Lipids	Ash	TDF^2^	SDF^3^	IDF^4^	Carbohydrates^5^
CF	10.3^a^ ± 0.1	13.5^c^ ± 2.4	1.6^c^ ± 0.3	2.1^d^ ± 0.0	4.2^c^ ± 0.2	0.5^c^ ± 0.2	3.7 ± 0.1	82.8
CPC (unsoaked)	8.4^b^ ± 0.1	54.6^b^ ± 0.2	2.8^b^ ± 0.3	6.4^b^ ± 0.1	16.4^b^ ± 0.4	2.2^b^ ± 0.4	14.2 ± 0.1	36.2
CPC‐S (soaked)	4.1^d^ ± 0.3	55.9^b^ ± 2.1	4.8^a^ ± 0.7	4.7^c^ ± 0.0	23.8^a^ ± 0.6	2.9^a^ ± 0.6	20.9 ± 0.1	34.7
CPI	5.3^c^ ± 0.0	87.5^a^ ± 2.6	0.8^d^ ± 0.1	6.5^a^ ± 0.0	3.6^c^ ± 0.1	3.2^a^ ± 0.1	0.4 ± 0.1	5.1

*Note*: Different letters “a, b” within the same row are statistically different.

Abbreviations: CF, cowpea flour; CPC, cowpea protein concentrate; CPC‐S, cowpea protein concentrate after soaking; CPI, cowpea protein isolate.

^1^ Results are expressed as % (d.b.), except for moisture, presented on a fresh weight basis.

^2^ Total dietary fiber.

^3^ Soluble dietary fiber.

^4^ Insoluble dietary fiber.

^5^ Total carbohydrates (including fiber). Means ± SD (*n* = 3).

Residual carbohydrates in CPC (36.2%) may be attributed to dietary fiber and incompletely separated starch, as dry fractionation methods have limited ability to separate protein‐rich particles from starch granules and cell wall material, especially in legume seeds. This incomplete separation occurs because protein bodies and starch granules often overlap in particle size and density, hindering complete partitioning into distinct fractions (Pelgrom et al. 2013; Pelgrom et al. 2015a; Pelgrom et al. 2015c). Other nutrients, including minerals, lipids, and TDF, can also be retained with protein‐rich portions (Rivera et al. 2024; Skylas et al. 2023). Moreover, CPC‐S had no significant impact on protein (*p* > 0.05) but led to relative enrichment in lipids and fiber, counterbalancing the solubilization of hydrophilic constituents (e.g., carbohydrates and minerals) in the soaking water. A similar trend was reported in carioca beans, where lipid content increased by approximately 50% from whole bean flour (4.6%) to protein concentrate (8.3%) (Santos et al. 2024).

Using the CPC as a feedstock input in a hybrid route (dry fractionation followed by wet extraction) yielded a CPI with protein content exceeding 80%, a yield of 45.0%, and a protein recovery of 62.3%, while markedly reducing carbohydrate and lipid content (Table 1). These outcomes align with the literature, which shows that hybrid approaches can combine the sustainability benefits of dry fractionation with the higher purity achievable by aqueous extraction, typically increasing protein recovery while reducing water use relative to full wet fractionation when optimized (Avila Ruiz et al. 2016). The predominance of soluble dietary fiber (SDF) over insoluble dietary fiber (IDF) following the hybrid process is noteworthy (Table 1), as pulses commonly exhibit higher IDF fractions. This shift is primarily attributed to the alkaline extraction stage, where the high pH promotes partial solubilization of cell wall pectins and hemicelluloses. Exposure to alkaline media disrupts intermolecular interactions within the cell wall matrix, increasing the extractability of these polysaccharides (Gao et al. 2020; Mapalagama et al. 2026). Additionally, centrifugation separates insoluble structural components, further concentrating the soluble fiber fraction in the final isolate and thereby increasing the water‐soluble fiber content.

### 3.2 Mineral and Metal Quantification

Minerals, such as calcium (Ca), iron (Fe), and zinc (Zn), are essential micronutrients, especially in plant‐based diets, where inhibitory compounds may limit mineral bioavailability. The elemental mineral and metal composition of cowpea protein ingredients is shown in Table 2. Protein isolation maintained comparable levels of Ca and Fe in CPI relative to CPC but reduced Zn content by 7%. However, the elevated ash content of CPI (∼6%) reflects the formation of inorganic salts, predominantly sodium chloride (NaCl), resulting from neutralization with NaOH and HCl. The observed NaCl content (>60 g kg^−1^, expressed as NaCl equivalent) can be attributed to salt retention in the protein matrix, as no washing or desalting step was performed after precipitation.

**TABLE 2:** Elemental mineral composition, heavy metals of cowpea protein ingredients, and reference intake values.

Ingredient	Element (mg kg^−1^)							NaCl^a^ (g kg^−1^)
	Cd	Pb	As	Cu	Ca	Fe	Zn	
**CPC**	<0.005	<0.005	<0.001	5.5	513.6	39.7	59.0	0.85
**CPI**	<0.005	<0.005	<0.001	7.2	565.9	41.4	54.6	60.9
**Daily limit (mg day^−1^)** ^b^	0.06	NA^c^	0.2^d^	35.0^e^	3000	NA^f^	45	<2000

Abbreviations: As, arsenic; Ca, calcium; Cd, cadmium; CPC, cowpea protein concentrate; CPI, cowpea protein isolate; Cu, copper; Fe, iron; NA, not applicable; Pb, lead; Zn, zinc.

^a^ NaCl expressed as sodium chloride equivalent, estimated as total salt content.

^b^ Reference intake values correspond to tolerable daily intake levels established by the Joint FAO/WHO Expert Committee on Food Additives (JECFA), considering a 70 kg adult.

^c^ No tolerable intake established by JECFA because a threshold could not be identified.

^d^ Benchmark dose lower.

^e^ Provisional maximum tolerable daily intake.

^f^ No maximum dose defined.

Residual salt levels in plant‐based ingredients depend strongly on processing conditions, particularly the extent of post‐precipitation purification. In conventional AE‐IEP routes, neutralization is a primary source of salt incorporation, and its removal depends on the efficiency of subsequent washing or membrane‐based operations (Mondor et al. 2010). For instance, significantly lower NaCl contents (12.0 g kg^−1^) have been reported for soy protein isolates (Peng et al. 2023), where a prolonged dialysis step (72 h) was applied. To mitigate these high sodium levels in future food applications, strategies, such as replacing NaOH with KOH, have been proposed to reduce sodium while increasing potassium content in the final product (Peng et al. 2023). This approach may improve nutritional profiles, given the recommended daily potassium intake (>3510 mg day^−1^). In addition, the type of acid used during isoelectric precipitation can influence the resulting salt composition, with organic acids (e.g., citric or lactic acid) reported to modify ionic strength and mineral profiles in protein isolates (Boye et al. 2010; Stone et al. 2015).

Moreover, post‐processing strategies, such as repeated washing, ultrafiltration with diafiltration (UF/DF), or dialysis, have been shown to reduce residual salt content in plant‐based protein isolates (Brião et al. 2024; Shrimant et al. 2024; Yaputri et al. 2023). From an industrial perspective, UF/DF represents the most viable option for salt removal, as membrane processes are already well established at pilot and industrial scales for the concentration and desalting of dairy and pulse protein ingredients. These systems allow continuous or semi‐continuous operation, efficient removal of low‐molecular‐weight compounds, and improved process control (Charcosset 2021; Mondor et al. 2009; Reig et al. 2021). Conversely, prolonged dialysis is generally restricted to laboratory‐scale applications due to its batch operation, long processing times, and high‐buffer consumption. Similarly, repeated washing of protein curds, although feasible, is typically associated with high water demand and lower efficiency in controlling mineral removal at a large scale (Ehsani et al. 2024; Hansen et al. 2022). It should also be noted that extensive salt removal may entail trade‐offs in protein yield, functionality, or process cost, requiring optimization based on the intended application (Boye et al. 2010; Mondor et al. 2010).

Heavy metals, including cadmium (Cd), lead (Pb), arsenic (As), and copper (Cu), were quantified in CPC and CPI to assess potential toxicological risks posed by these ingredients. Cd and Pb concentrations were below the analytical detection limit (5 µg kg^−1^), and As was inferior to 1 µg kg^−1^ for both cowpea‐derived ingredients, complying with the regulatory standards established for legumes (Brazil 2013). Cu content increased by approximately 30% in the CPI relative to CPC (Table 2), yet remaining within the tolerable daily intake of 0.5 mg kg^−1^ of body weight (equivalent to 35 mg day^−1^ for a 70 kg adult) established by the FAO/WHO (1982). Overall, our results align with the mineral composition of pulses from other regions. Beans from China exhibited average concentrations of As (0.03 mg kg^−1^), Cd (0.04 mg kg^−1^), and Pb (0.02 mg kg^−1^), remaining below health risk thresholds (Yu et al. 2023). Salama and Radwan (2005) reported Cd and Pb levels of 0.01 and 0.13 mg kg^−1^, respectively, for Egyptian cowpeas, with higher concentrations of Cu (3.17 mg kg^−1^) and Zn (12.45 mg kg^−1^). The low heavy metal content of cowpea‐derived ingredients eliminates toxicological risks and ensures food safety, minimizing long‐term health risks associated with chronic metal exposure (Abubakar et al. 2024).

### 3.3 The Structural Properties of Cowpea Ingredients

The structural organization and morphology of cowpea ingredients (CPC, CPC‐S, and CPI) were examined using SEM (including CF), SDS–PAGE, FTIR, and DSC analyses, providing complementary insights into how fractionation methods and post‐processing treatments influence protein configuration at the molecular and supramolecular levels.

**FIGURE 2:**
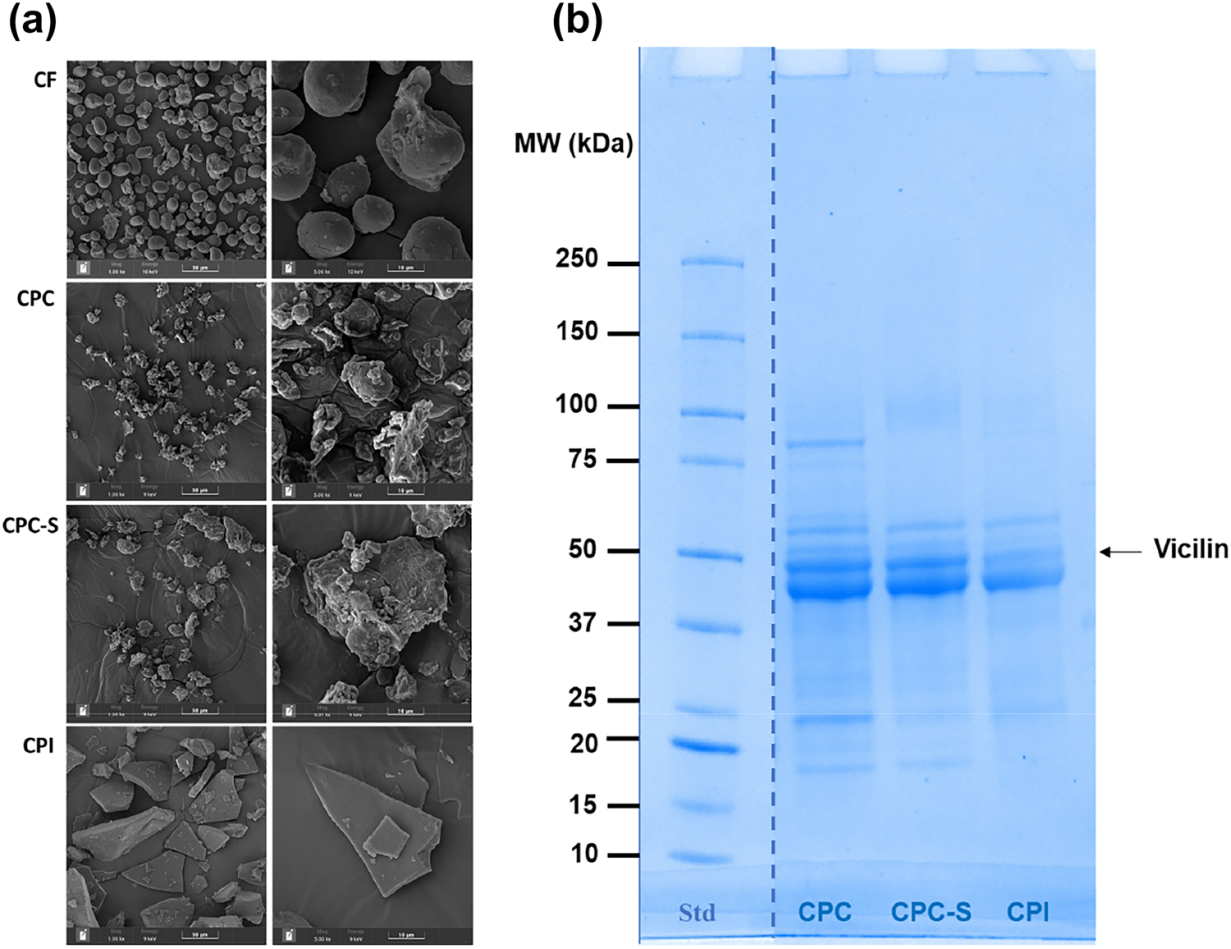
(a) Representative SEM images of cowpea ingredients (CF, CPC, CPC‐S, and CPI), (b) SDS–PAGE profile of cowpea protein ingredients (CPC, CPC‐S, and CPI). (a) Scanning electron microscopy (SEM) images were acquired at 1000× (scale bar = 50 µm; left) and 5000× magnification (scale bar = 10 µm; right); (b). The dashed line indicates the removal of intervening lanes containing unrelated samples for clarity. All displayed lanes originated from the same gel and were subjected to identical electrophoretic and staining conditions. CPC, cowpea protein concentrate; CPC‐S, cowpea protein concentrate (soaked); CPI, cowpea protein isolate; MW, molecular weight marker; Std, standard.

SEM images (Figure 2a) revealed clear morphological differences among the ingredients. CF exhibited a microstructure dominated by smooth, ellipsoidal starch granules (∼10–30 µm), consistent with typical pulse cotyledon morphology (Pelgrom et al. 2013; Schutyser and van der Goot 2011). Smaller, irregular fragments adhered to the granule surfaces were observed, likely corresponding to protein bodies and cell wall residues and indicative of incomplete cellular disruption during dry processing, resulting in particles containing both starch and protein (Pelgrom et al. 2013; Skylas et al. 2023).

In contrast, the dry‐fractionated protein concentrate (CPC) showed irregular, rough‐surfaced particles with a broad size distribution, indicating protein enrichment and partial starch removal during air classification. Although some residual rounded starch granules remained visible, most particles appeared as fragmented aggregates. Such heterogeneous aggregates of protein, starch, and fiber are characteristic of air‐classified pulse fractions, where overlap in size and density between starch granules and protein bodies limits complete separation (De Angelis et al. 2024; Fernando 2021; Pelgrom, Wang, et al. 2015c; Pelgrom, Boom, et al. 2015b). After soaking and freeze‐drying, CPC‐S exhibited a more agglomerated, expanded structure than CPC, with larger, denser, and more irregular surface particles, indicating structural reorganization. Soaking promotes swelling of the protein–polysaccharide complex and partial leaching of soluble components, whereas freeze‐drying induces ice‐crystal formation followed by sublimation, leading to structural rearrangement of particles (Brishti et al. 2020). Together, these results indicate that soaking primarily affects protein aggregation, intermolecular interactions, and the organization of protein–polysaccharide complexes, rather than protein primary or secondary structure.

The protein isolate (CPI) exhibited markedly different morphology, with flat, plate‐like particles with smooth surfaces and angular edges, forming lamellar structures. This morphology has been reported as typical of freeze‐dried CPIs and has been attributed to protein aggregation and reorganization during AE‐IEP and drying (Loushigam and Shanmugam 2023; Zolqadri and Li 2025). The absence of discrete starch granules and the formation of a continuous protein‐rich matrix suggest a high degree of protein–protein interaction, which corroborates the higher protein purity of CPI. These results align with those of Hamed et al. (2024), who observed that a mixture of pulse proteins (faba bean, soybean, and lupine) exhibited an irregular surface microstructure, suggesting the formation of a dense, cohesive protein network during isolation.

The SDS–PAGE profiles under reducing conditions showed a broad distribution of protein subunits spanning ∼18–80 kDa (Figure 2b), reflecting the complex protein composition of cowpea seeds. The most intense bands across all cowpea protein ingredients were observed in the ∼45–60 kDa region, corresponding to the MW range of dissociated vicilin‐type (7S) globulin subunits, the major storage proteins in *V. unguiculata* (Vasconcelos et al. 2010; Zolqadri and Li 2025). The persistence of these bands across all ingredients shows that the major storage protein fractions were preserved regardless of the recovery process. However, differences in band distribution and intensity reflect the effects of processing on protein fractionation.

CPC and CPC‐S exhibited more complex electrophoretic profiles, with additional bands in the ∼18–30 kDa region. Under reducing conditions, these bands are associated with low‐MW protein fractions, including basic legumin (11S) subunits and albumin (Freitas et al. 2004; Vasconcelos et al. 2010). The presence of these bands suggests that dry fractionation preserves a broader spectrum of native protein components, including soluble and minor fractions that may be partially removed during wet extraction. The similarity between CPC and CPC‐S profiles indicates that the soaking treatment did not significantly alter the protein's primary structure. Minor differences in band sharpness and intensity, especially in the lower MW region, may reflect partial leaching of water‐soluble proteins or structural rearrangements induced by hydration and subsequent freeze‐drying. These results align with SEM observations, which showed that CPC and CPC‐S formed heterogeneous, aggregated structures composed of protein, starch, and fiber. Conversely, CPI showed a uniform, lamellar structure typical of protein‐rich matrices.

In contrast, CPI showed a simplified profile with higher relative intensity in the ∼40–55 kDa region and reduced contributions from lower MW bands. This pattern indicates selective enrichment of vicilin‐type globulins, consistent with the wet extraction, which favors solubilization and recovery of globulin fractions while partially reducing the relative contribution of more soluble or weakly associated proteins (Vogelsang‐O'Dwyer, Bez, et al. 2020; Zolqadri and Li 2025). A faint band near ∼37 kDa was observed only in CPI and may correspond to an acidic legumin subunit or a vicilin‐derived fragment; however, given its low intensity, this assignment should be considered cautiously.

FTIR analysis of the Amide I region (1600–1700 cm^−^^1^), along with Gaussian deconvolution (Figure S3), provided additional evidence supporting these results, revealing subtle yet consistent differences in protein secondary structure. To minimize overparameterization and ensure cross‐sample comparability, a constrained fitting approach was employed, as given the high sensitivity of the amide I region to spectral overlap and baseline artifacts (Barth 2007; Kauppinen et al. 1981; Sadat and Joye 2020). All protein ingredients primarily consisted of unordered structures (random coil) and β‐turns, with fewer β‐sheet structures. CPC showed that 50.0% was random coil, 41.5% were β‐turns, and only 8.5% were β‐sheet. CPC‐S showed a similar distribution: 49.2% random coil, 41.7% β‐turns, and 9.1% β‐sheet, suggesting that soaking did not cause significant changes in the secondary‐structure profile under the conditions employed.

In contrast, CPI showed a slightly higher contribution from β‐sheet structures (∼15%), along with modest reductions in random coil and β‐turns compared to the dry‐fractionated ingredients. The structural shift suggests a partial rearrangement of the protein conformation caused by AE‐IEP, which can promote partial unfolding and molecular reorganization, as the formation of intermolecular β‐sheet structures (Tang 2008; Teixeira et al. 2024).

These structural differences were reflected in the thermal behavior of CPC, CPC‐S, and CPI, as observed by DSC (Figure S4). CPC exhibited two endothermic transitions at 147.4°C and 192.5°C, with associated enthalpies (∆*H*) of 40.3 and 54.3 J g^−1^, respectively, indicating the presence of thermally distinct domains within a heterogeneous matrix. The lower temperature event can be associated with the main protein‐rich fraction. At the same time, the higher temperature transition likely reflects more stable structures arising from protein–carbohydrate interactions or strongly aggregated domains. Multiple transitions have been observed in pulse protein systems and reflect matrix complexity rather than single‐protein unfolding events (Hamed et al. 2024; Ricci et al. 2018).

CPC‐S showed a single transition at 156.6°C with a lower enthalpy (Δ*H* = 14.3 J g^−1^), suggesting fewer native or ordered structures available for unfolding. This is consistent with SEM observations of increased particle agglomeration after soaking and freeze‐drying and consistent with SDS–PAGE and FTIR results indicating that primary and secondary structures are preserved, but intermolecular organization is altered. Similarly, CPI exhibited a single transition at 155.5°C with the lowest enthalpy (Δ*H* = 11.2 J g^−1^), indicating a reduced availability of ordered structures. Although achieved under relatively mild conditions (pH 9.0, 50°C), the combined effects of AE‐IEP and freeze‐drying induced conformational changes. According to Long et al. (2025), exposure to alkaline conditions and approaching the IEP increases electrostatic repulsion, promoting protein unfolding and subsequent re‐aggregation. This evidence is supported by increased β‐sheet content observed by FTIR and the lamellar morphology in SEM, both of which are indicative of protein aggregation and structural reorganization.

The progressive decrease in ∆*H* from CPC to CPC‐S and CPI indicates fewer ordered structures available for thermal transitions. This trend reflects the increasing degree of structural modification induced by the processing routes, ranging from dry fractionation to soaking and the AE‐IEP process. Importantly, the transition temperatures of protein ingredients (∼147–157°C) fall within the range reported for pulse protein powders. However, values can vary significantly depending on protein source, extraction conditions, moisture, and matrix interactions (Ricci et al. 2018). Overall, DSC results align with structural evidence from SEM, SDS–PAGE, and FTIR analyses, underscoring the importance of considering both compositional and conformational factors and demonstrating that the processing route influences not only protein composition but also structural organization across multiple scales. Dry fractionation preserves a heterogeneous structure with multiple interacting components, whereas soaking promotes structural reorganization without major conformational changes. On the other hand, the hybrid extraction process leads to a more compact, protein‐rich matrix characterized by molecular rearrangement. These factors are considered when evaluating the techno‐functional and thermal properties of pulse protein ingredients.

### 3.4 Amino Acid Profile, IVPD, and IV‐PDCAAS

The amino acid profiles of CPC and CPI (Table 3) were consistent with those reported for different cowpea cultivars (Shevkani et al. 2025; Teka et al. 2020). On a sample basis, CPI contained significantly higher total amino acids (70.7 g 100 g^−1^) and EAAs (31.0 g 100 g^−1^) than CPC (44.1 and 20.2 g 100 g^−1^, respectively), reflecting the higher protein purity achieved by the hybrid isolation process. Among the EAAs, leucine (Leu), lysine (Lys), valine (Val), isoleucine (Ile), and aromatic amino acids (tyrosine and phenylalanine) were the most abundant in both ingredients (Table 3). In particular, Leu, Ile, and Val confirmed cowpea as a relevant source of BCAAs, which are vital for muscle protein synthesis and metabolic regulation (Shevkani et al. 2025; Vasconcelos et al. 2010).

**TABLE 3:** Amino acid composition (g 100 g^−1^ sample) and amino acid score (AAS, %) of cowpea protein ingredients.

Essential amino acid (EAA)^1^	CPC (g 100 g^−1^ sample)	CPI (g 100 g^−1^ sample)	FAO/WHO requirements^2^	CPC (AAS, %)^3^	CPI (AAS, %)^3^
Histidine (His)	1.5^b^ ± 0.04	2.4^a^ ± 0.1	1.9	179.0^a^ ± 5.4	178.0^b^ ± 5.3
Isoleucine (Ile)	2.0^b^ ± 0.06	3.2^a^ ± 0.1	2.8	164.4^a^ ± 4.9	164.3^a^ ± 4.9
Leucine (Leu)	3.5^b^ ± 0.11	5.7^a^ ± 0.2	6.6	122.3^a^ ± 3.8	122.0^a^ ± 3.7
Lysine (Lys)	3.1^b^ ± 0.09	4.8^a^ ± 0.1	5.8	123.1^a^ ± 3.7	116.1^b^ ± 3.5
Threonine (Thr)	1.7^b^ ± 0.05	2.4^a^ ± 0.1	3.4	113.4^a^ ± 3.4	100.3^b^ ± 3.0
Tryptophan (Trp)	0.7^b^ ± 0.02	1.1^a^ ± ± 0.0	1.1	136.0^b^ ± 4.1	142.8^a^ ± 4.3
Valine (Val)	2.3^b^ ± 0.07	3.5^a^ ± 0.1	3.5	150.9^a^ ± 4.5	141.1^b^ ± 4.2
Sulfur total amino acids (Met + Cys)	0.9^b^ ± 0.03	1.4^a^ ± 0.0	2.5	79.8^a^ ± 2.4	80.4^a^ ± 2.3
Aromatic total amino acids (Phe + Tyr)	4.3^b^ ± 0.13	6.6^a^ ± 0.2	6.3	156.5^a^ ± 4.7	147.1^b^ ± 4.4

Nonessential amino acid (NEAA)^1^	CPC (g 100 g^−1^ sample)	CPI (g 100 g^−1^ sample)
Aspartic acid (Asp)	5.0^b^ ± 0.15	9.8^a^ ± 0.3
Glutamic acid (Glu)	7.8^b^ ± 0.24	12.7^a^ ± 0.4
Alanine (Ala)	1.9^b^ ± 0.06	2.9^a^ ± 0.1
Arginine (Arg)	3.4^b^ ± 0.10	4.8^a^ ± 0.1
Glycine (Gly)	1.7^b^ ± 0.05	2.7^a^ ± 0.1
Proline (Pro)	1.8^b^ ± 0.05	2.9^a^ ± 0.1
Serine (Ser)	2.2^b^ ± 0.07	3.9^a^ ± 0.1
**Total EAAs**	20.2^b^ ± 0.60	31.0^a^ ± 0.9
**Total NEAAs**	23.9^b^ ± 0.72	39.7^a^ ± 0.7
**Total amino acids** ^1^	44.1^b^ ± 1.32	70.7^a^ ± 1.3

*Note*: Different letters “a, b” in the same line indicate significant differences (*p* < 0.05). Values are means ± standard deviation.

Abbreviations: CPC, cowpea protein concentrate; CPI, cowpea protein isolate; Cys, cysteine; Met, methionine; Phe, phenylalanine; Tyr, tyrosine.

^1^ Amino acid composition expressed as g 100 g^−1^ sample.

^2^ FAO/WHO (1991) reference pattern for preschool‐aged children (2–5 years), expressed as g 100 g^−1^ protein.

^3^ Amino acid score (AAS) calculated using amino acid contents normalized to protein content.

In contrast, SAAs (methionine and cysteine) were the least abundant EAA group, confirming their role as the limiting amino acid group in cowpea proteins, as commonly reported for legumes and pulses (Vasconcelos et al. 2010; Zolqadri and Li 2025). Although SAAs may undergo partial degradation during acid hydrolysis, all samples were analyzed under identical conditions, ensuring the validity of the comparative interpretation. Non‐EAAs (NEAAs), primarily aspartic acid (Asp) and glutamic acid (Glu), accounted for approximately 55% of total amino acids (Table 3) and play fundamental roles in gut health and antioxidant defense (Shevkani et al. 2025; Teka et al. 2020).

The IVPD values for CPC and CPI were 82.5% ± 0.8% and 85.8% ± 1.1%, respectively, approaching the upper range reported for cowpea varieties (67.7%–84.9%) (Kalpanadevi and Mohan 2013; Shevkani et al. 2025; Teka et al. 2020). Differences in IVPD among cowpea‐derived ingredients may be attributed to genotype, macronutrient composition, processing and extraction route, environmental conditions, and residual ANFs. The AAS for both CPC and CPI met or exceeded the FAO/WHO (1991) reference pattern for preschool‐aged children (2–5 years old) for all EAAs, except limiting SAAs. The calculated IV‐PDCAAS values were 65.8% ± 1.6% for CPC and 69.9% ± 2.2% for CPI, exceeding those reported for lentil protein concentrate (55.9%), faba bean (55.2%), and pea flour (52.1%) (Nosworthy and House 2017; Stone et al. 2019). These results indicate that the hybrid route improved protein purity and digestibility while maintaining a favorable profile of indispensable amino acids.

### 3.5 Antinutritional Factors

The ANFs (phytates, trypsin inhibitors, and tannins) were quantified in CPC (unsoaked and soaked CPC) and CPI to evaluate the effects of soaking and protein isolation on their retention (Table 4). CPC‐S significantly (*p* < 0.05) reduced the contents of all evaluated ANFs compared to untreated CPC, with the greatest decreases in phytates (70%) and total tannins (49%), and a slight decrease (11%) in TIA. Soaking is an efficient method for removing water‐soluble and diffusible compounds, such as phytic acid and soluble phenolics (Ravoninjatovo et al. 2022). On the other hand, trypsin inhibitors are insoluble compounds in water, often forming stable protein–polysaccharide complexes, which makes them less susceptible to removal under mild aqueous conditions (Egounlety and Aworh 2003; Kalpanadevi and Mohan 2013; Khattab and Arntfield 2009) and temperatures below 90°C (Yang et al. 2024).

**TABLE 4:** Antinutritional factors of cowpea protein concentrate (unsoaked and soaked) and the protein isolate.

Ingredient	Phytates (mg PAE g^−1^)	TIA (TIU mg^−1^)	Total tannins (mg TAE g^−1^)
CPC	2.0^a^ ± 0.02	5.4^a^ ± 0.2	26.1^b^ ± 0.02
CPC‐S	0.6^b^ ± 0.01	4.8^c^ ± 0.1	13.2^c^ ± 0.01
CPI	1.9^a^ ± 0.01	5.1^b^ ± 0.2	29.8^a^ ± 0.01

*Note*: Means ± standard deviation. Means with different letters “a, b” in the same row are significantly different (*p* < 0.05).

Abbreviations: PAE, phytic acid equivalents; TAE, tannic acid equivalents; TIA, trypsin inhibitor activity; TIU, trypsin inhibition units.

When comparing the ANF content of CPI to unsoaked CPC, TIA showed a slight decrease (5%), whereas phytate content remained unchanged (*p* > 0.05), and tannin content increased by 14%. These results suggest that ANFs are tightly bound to the protein fraction of dry‐fractionated CPC even after purification by wet extraction. For example, the higher tannin content in CPI could result from the co‐extraction of phenolic compounds bound to proteins during water solubilization (at pH 9.0) and their subsequent co‐precipitation at IEP (pH 4.5), as reported for pea, kidney bean, and faba bean protein isolates (Shevkani et al. 2015). Moreover, the higher SDF levels in CPI (Table 1) may contribute to tannin retention via hydrophilic and hydrogen‐bonding interactions (Jakobek 2015; Renard et al. 2017).

Protein‐like ANFs, such as trypsin inhibitors, can be retained in CPI when protein is concentrated (Dumoulin et al. 2021) and no specific thermal or enzymatic inactivation pretreatments are applied. Protease inhibitors exhibit high structural stability, particularly those of the Kunitz type found in legumes (Mubarak 2005). Therefore, additional targeted processing, such as controlled heat treatment, cooking, fermentation, or enzymatic hydrolysis, would be required during the wet purification extraction stage of the hybrid route to achieve a substantial reduction in phytates, tannins, and protease inhibitors in the CPI without compromising functional properties. Alternatively, a thermal–mechanical posttreatment like extrusion, a well‐established technology for ANF reduction across various plant ingredients, could be applied either to the dewatered (offering energy savings by circumventing full dehydration and rehydration) or to the final dried concentrate or isolate, achieving similar reductions without compromising functional properties.

### 3.6 Techno‐Functional Properties of Cowpea Proteins

**FIGURE 3:**
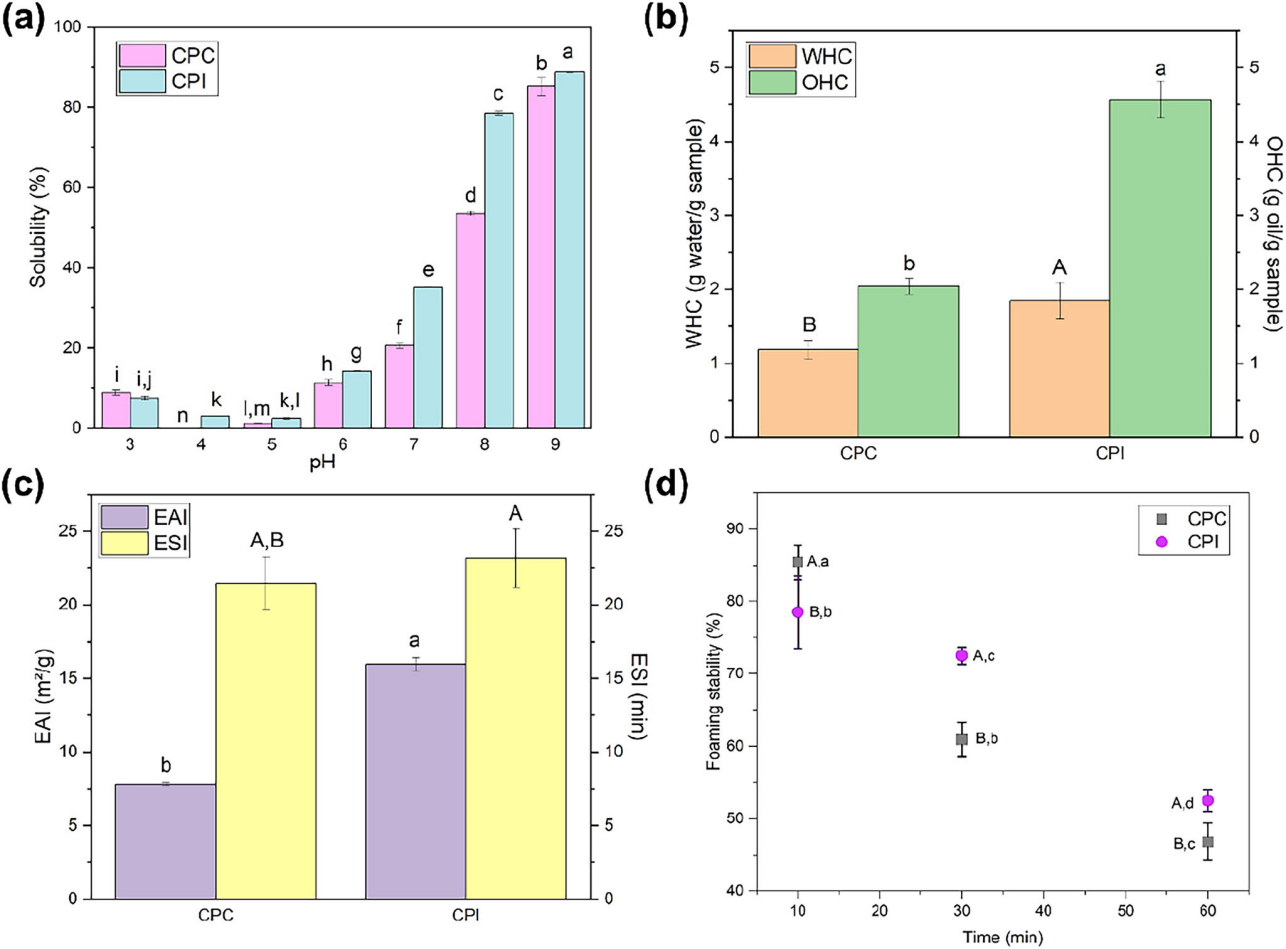
(a) Effect of pH on solubility, (b) water‐ and oil‐holding capacity, (c) emulsifying properties, and (d) foaming stability of cowpea protein ingredients (CPI and CPC). ^a,b^Different letters indicate significant differences (*p* < 0.05) within each parameter. CPC, cowpea protein concentrate; CPI, cowpea protein isolate; EAI, emulsifying activity index; ESI, emulsion stability index; OHC, oil‐holding capacity; WHC, water‐holding capacity.

**FIGURE 4:**
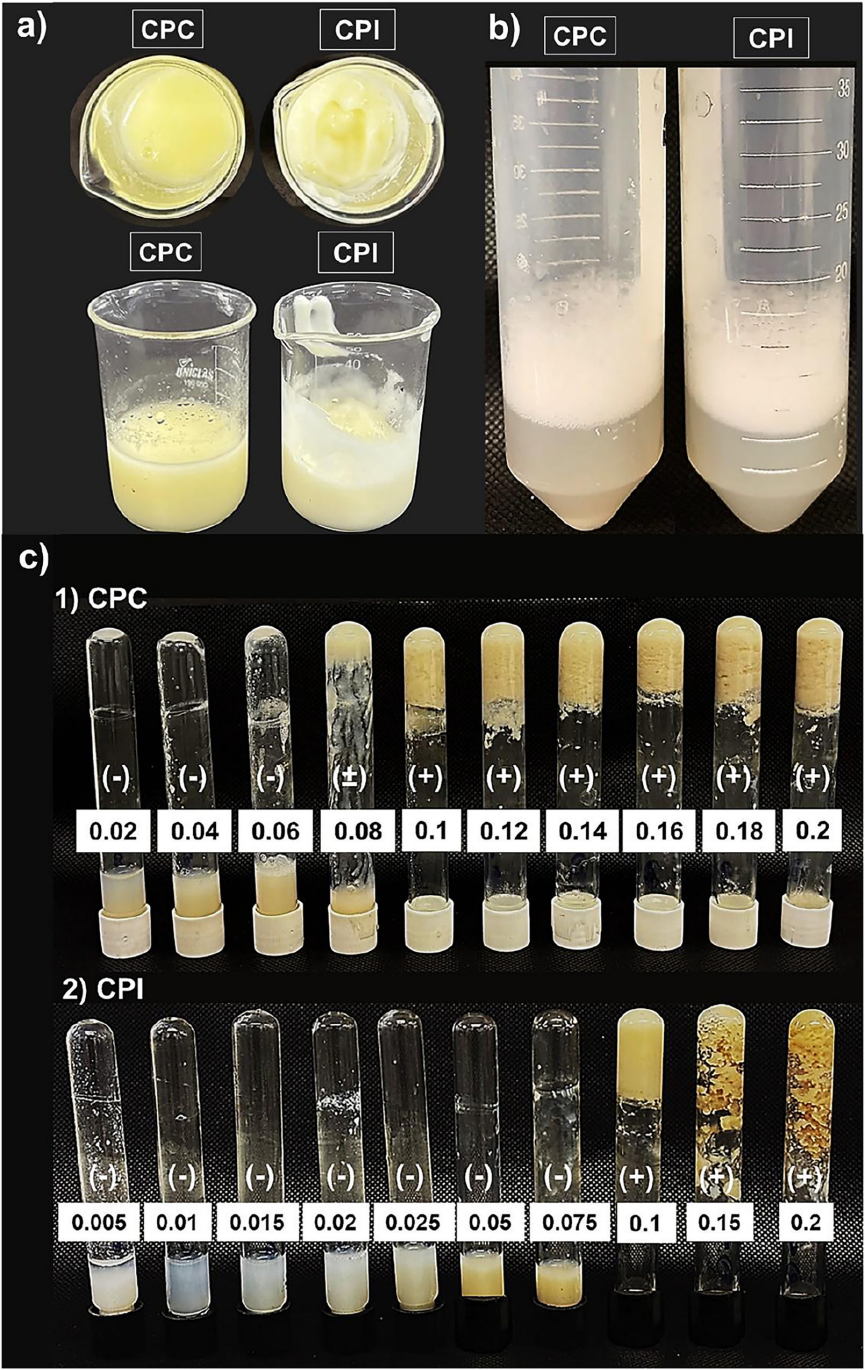
Visual appearance of cowpea protein concentrate (CPC) and cowpea protein isolate (CPI): (a) emulsions, (b) foams, and (c) gel formation at increasing protein concentrations. Protein concentrations ranged from 0.020 to 0.20 mg mL^−1^ for CPC, and from 0.005 to 0.20 mg mL^−1^ for CPI. (−) No gel formation; (±) weak gel; and (+) gel formation.

The techno‐functional properties CPC and CPI were evaluated in terms of solubility, water‐ and oil‐holding capacities, EAI and ESI, FC and FS, and gel‐forming ability (Figures 3 and 4).

The solubility profiles of CPC and CPI from pH 3.0 to 9.0 are shown in Figure 3a. CPI exhibited higher solubility than CPC in all pH conditions, with both ingredients reaching minimal solubility (<2%) between pH 4.0 and 5.0, corresponding to the protein IEP as observed by Ragab et al. (2004) for cowpea proteins. As the pH increased, both CPC and CPI showed significant increases in solubility, reaching ∼90% at pH 9.0. At IEP, proteins have no net charge, leading to reduced electrostatic repulsion and, consequently, precipitation (Singhal et al. 2016). As pH increases, the ionization of acidic groups increases negative charge and repulsion, thereby enhancing solubility (Boye et al. 2010; Shevkani et al. 2015). De Angelis et al. (2024) reported that dry‑fractionated pulse protein concentrates can exhibit higher solubility than protein obtained by the wet extraction method. This result may be attributed to the preservation of native protein conformation during dry fractionation processing, which avoids denaturation and aggregation often induced by pH extremes and ionic shifts in wet extraction. In the present study, the hybrid route (dry + wet fractionation) produced CPI with enhanced solubility across the tested pH values, indicating the removal of insoluble components.

Protein's water‐ and oil‐holding capacities (WHC and OHC) are key parameters that dictate protein functionality in complex food systems, affecting the texture, juiciness, and stability of formulated products (Ge et al. 2021; Shevkani et al. 2015). These attributes are governed by the exposure and balance between hydrophilic and hydrophobic groups in the protein structure, as well as its conformational flexibility (Ma, Greis, et al. 2022; Shevkani et al. 2015). The WHC and OHC of cowpea protein ingredients are shown graphically in Figure 3b.

CPI exhibited an OHC of 4.6 g oil g^−1^, twice that of the CPC (2.0 g oil g^−1^), and higher than values reported for cowpea (1.1, 0.85 g oil g^−1^) and soy (1.2 g oil g^−1^) protein isolates (Ragab et al. 2004; Witono et al. 2014). This difference can be attributed to compositional and structural factors. The higher protein content in CPI likely increased exposure of hydrophobic side chains and tertiary‐structure unfolding, thereby favoring lipid entrapment, whereas matrix components in CPC may limit this interaction. This feature is especially desirable in high‐fat meat product analogs, where lipid retention improves flavor release and contributes to a juicy mouthfeel (Alfaro‐Diaz et al. 2021).

Regarding WHC, legume protein isolates typically exhibit values ranging from 1.2 to 6.8 g water g^−1^ (Gundogan and Can Karaca 2020; Ma, Grossmann, et al. 2022). CPI also showed improved WHC (1.8 g water g^−1^) over CPC (1.2 g water g^−1^, Figure 3b). This behavior can be attributed to differences in composition and protein structure, including CPI's higher protein content and partial unfolding with disruption of non‐covalent bonds during the wet extraction process. These factors likely increase the exposure of polar groups that can bind water (Ma, Greis, et al. 2022; Schlangen et al. 2022). In contrast, CPC contains a higher proportion of nonprotein components, such as starch and fiber, which contribute to water retention through matrix effects but may exhibit lower binding capacity than protein‐rich systems (Schlangen et al. 2022). Moreover, structural features, such as protein conformation and net surface charge, modulate water‐binding in high‐protein systems with reduced polysaccharide content (Lafarga et al. 2018; Schlangen et al. 2022). High WHC is technologically relevant for enhancing succulence and softness in meat analogs and for contributing to viscosity in semi‐solid systems. In extrusion‐based texturized vegetable protein (TVP) production, WHC is a critical determinant of porosity and air cell structure, directly affecting texture perception in the final product (Ma, Greis, et al. 2022; Samard and Ryu 2019).

The EAI and ESI of both cowpea protein ingredients are presented in Figure 3c. CPI exhibited an EAI of 15.9 m^2^ g^−1^, approximately twice that of CPC (7.8 m^2^ g^−1^). As EAI is expressed on a protein basis, this difference reflects intrinsic differences in protein functionality, including improved solubility and enhanced interfacial activity. The isolate's higher protein content facilitates faster adsorption and polypeptide chain alignment at the water–oil interface. The found values are comparable to the emulsifying properties of common beans (11.8–22.3 m^2^ g^−1^) (Gundogan and Can Karaca 2020; Shevkani et al. 2015). However, ESI values were statistically similar (CPI: 23 min vs. CPC: 21 min; *p* > 0.05), despite CPI showing slightly better emulsion stability than CPC. De Angelis et al. (2024) reported that the association of proteins with fibrous and starch components in dry‐fractionated pulse proteins can elevate viscosity and contribute to emulsion stabilization despite lower EAI. Still, for CPI, emulsion stabilization mainly relies on the proteins’ surface charge, solubility, molecular flexibility, and conformational structure to form a cohesive interfacial layer (Gundogan and Can Karaca 2020; Pearce and Kinsella 1978; Shevkani et al. 2015). This background supports the observation that CPC, despite lower protein purity, may still perform well in emulsification when the protein structure is preserved, especially under mild processing conditions.

Foaming properties are relevant for protein applications in food systems, especially in products that require aeration, such as ice creams, mousses, whipped toppings, cakes, and meringues (Adebiyi and Aluko 2011). The results for FC (*t* = 0 min) of cowpea protein ingredients showed that CPI (70.0%) had a higher FC than CPC (53.3%), likely due to the removal of nonprotein material and structural reorientation during the hybrid extraction process. Regarding FS (measured from 10 to 60 min), CPC initially retained foam volume more effectively (85.4% at 10 min) than CPI (78.5%, Figure 3d), suggesting that the nonprotein matrix in CPC (especially polysaccharides and fiber) may enhance viscosity and slow bubble drainage, improving short‐term stability. According to De Angelis et al. (2024), dry‐fractionated proteins can also exhibit higher foaming capacities than wet‐extracted pulse proteins. However, after 60 min, CPI maintained foam integrity better (52.5% vs. 46.8%, *p* < 0.05), attributed to stronger, more cohesive protein films and improved solubility at the foaming pH (7.0).

Overall, the distinct FC and FS profiles observed for CPC and CPI highlight their complementary roles in aerated food systems. Dry‐fractionated concentrates, such as CPC, which retain starch and fiber, appear to stabilize the aerated matrix at early stages, likely through increased viscosity and reduced liquid drainage (De Angelis et al. 2024; Schlangen et al. 2022). Conversely, CPI produced by hybrid routes can develop stronger interfacial networks over time, attributed to its higher protein purity and solubility at pH 7.0. These attributes facilitate the formation of cohesive, elastic interfacial films required to stabilize air bubbles for extended periods and prevent coalescence (Adebiyi and Aluko 2011; Mundi and Aluko 2012; Shevkani et al. 2015). On the basis of these complementary techno‐functional characteristics, the combined use of CPC and CPI may represent a promising strategy to modulate aeration performance in formulated foods by integrating rapid film formation with matrix‐mediated stabilization.

Gelling capacity is a critical techno‐functional property in plant‐based protein applications, such as meat analogs, puddings, and gel‐based desserts (Costa et al. 2025; Ma, Greis, et al. 2022), where elasticity, cohesiveness, and moistness are key to consumer acceptability. Gelation happens when proteins form a network that resists flow (Boye et al. 2010). The results of the qualitative analysis of the gel‐forming ability of CPC and CPI are shown in Figure 4. Visually observing the solutions allows for the assessment of the stability of the formed gels (liquid (−), viscous (±), or firm (+)). The least gelling concentration (LGC) is defined as the lowest protein concentration required to form a self‐supporting gel; the lower the LGC, the stronger the gelation ability (Gundogan and Can Karaca 2020). LGC was 0.1 g mL^−1^ (10% w/v) for both CPC and CPI, consistent with values reported for bean protein isolates (0.08–0.1 g mL^−1^) from different varieties (Costa et al. 2025; Gundogan and Can Karaca 2020). In comparison to other pulses, protein isolates from soybean, fava bean, pea, and lentil displayed slightly higher LGC (12%–15% w/v) (Ma, Grossmann, et al. 2022), but comparable to the LGC range for the pulse protein concentrates (8%–14% w/v) (Boye et al. 2010).

From a processing perspective, proteins obtained through dry fractionation (as in CPC) form gels and dispersions that contribute to the presence of starch and fiber (De Angelis et al. 2024). In contrast, for wet‐processed isolates (as in CPI), the structure strength relies on the protein itself due to the removal of co‐structuring nonprotein fractions. This functional divergence underscores the importance of selecting a protein recovery method tailored to the intended food application, particularly in structured products where the integrity of the protein network is crucial. Therefore, the choice among dry, wet, or hybrid methods must consider not only yield and environmental impact but also the specific targeted food application.

## 4 Conclusion

Dry fractionation is a robust, sustainable process that preserves the protein's native structure and can be incorporated to add value to underutilized pulses, such as cowpeas, thereby contributing to the diversification of plant‐protein sources. Dry‐fractionated CPC showed moderate protein enrichment and considerable fiber content, highlighting the need to optimize both the grinding and air‐classification steps for efficient scale‐up. The nutrient profile of dry‐fractionated CPC is preserved, and the soaking treatment reduced soluble antinutrients, suggesting improved nutritional accessibility without major structural alteration. Additionally, the retention of fiber and other native components in CPC results in a more complex ingredient matrix, which may confer formulation advantages by enhancing functional performance and reducing reliance on added ingredients.

Wet fractionation, conversely, enables targeted structural modifications and enhances nutritional and specific functionalities, as CPI demonstrated high‐protein purity and improved amino acid profile. However, the high sodium content of CPI after purification underscores the need to explore desalination strategies to minimize residual salt and align with nutritional guidelines for sodium intake. The technological evaluation of cowpea protein ingredients revealed enhanced interfacial and hydration‐related functionalities, including emulsifying, foaming, and gel‐forming properties, demonstrating competitive performance with pea, lentil, and soy protein ingredients reported in the literature. Therefore, hybrid routes for protein recovery integrate the environmental benefits of dry methods with the functional advantages of wet extraction. In conclusion, the selection of a dry, wet, or hybrid processing route should be determined on a fit‐for‐purpose basis, aligning the chosen process with the desired functional attributes, final application, target scale, and sustainability goals, offering a balanced approach that improves protein purity and functionality while reducing processing intensity compared to fully wet routes.

## Author Contributions

**Renata Fialho Teixeira**: conceptualization, investigation, funding acquisition, writing – original draft, methodology, validation, visualization, formal analysis, data curation, writing – review and editing, software. **Clóvis Antônio Balbinot Filho**: investigation, validation, writing – review and editing, methodology. **Jaíne Oliveira**: investigation, methodology, validation. **Graziele Grossi Bovi Karatay**: funding acquisition, writing – review and editing, project administration, visualization, resources. **Cristiana Ambiel**: funding acquisition, writing – review and editing, project administration, resources. **Acácio Antonio Ferreira Zielinski**: funding acquisition, resources, supervision, investigation, visualization, data curation.

## Conflicts of Interest

The authors declare no conflicts of interest.

## Supporting information




**Supplementary Material**: jfds71256‐sup‐0001‐SuppMat.docx
