# Supplementary material for: Hybrid Fractionation of Cowpea: Combining Dry and Wet Routes to Produce Versatile Protein Ingredients
Source: J Food Sci. 2026 Jul 13;91(7):e71256. doi: 10.1111/1750-3841.71256 (PMC13359296; doi:10.1111/1750-3841.71256)
Supplement: Supplementary file 1 — Supplementary Material: jfds71256‐sup‐0001‐SuppMat.docx [file JFDS-91-0-s001.docx]

**Hybrid fractionation of Cowpea: combining dry and wet routes to produce versatile protein ingredients**

Renata Fialho Teixeira^a,*^, Clóvis A. Balbinot Filho^a^, Jaíne Oliveira^a^, Graziele Grossi Bovi Karatay^b^, Cristiana Ambiel^b^, and Acácio A. F. Zielinski^a,*^

^a^Department of Chemical Engineering and Food Engineering, Federal University of Santa Catarina, Florianópolis, SC, 476, 88040-900, Brazil.

^b^ Science and Technology Department, The Good Food Institute Brasil, São Paulo, Brazil

# Supplementary material

The chemicals used are sodium hydroxide (P.A., Neon ®), hydrochloric acid (P.A., Êxodo Científica®), sulfuric acid (P.A., Neon®), acetic acid (P.A., Neon®), boric acid (P.A., Neon®), ethanol (99.5%, Êxodo científica®), n-hexane (99% P.A., Neon®), amylases (Termamyl 120L and AMG® 300L, Novozymes®), Folin-Ciocalteu phenolic solution (2M, 47641, Sigma®), Brilliant blue G-250 (Êxodo científica®), Bio-Rad Protein Assay kit I (#5000001, Bio-Rad®), BAPNA (Nα-Benzoyl-DL-arginine 4-nitroanilide hydrochloride, ≥ 98%, B4875, Sigma®), DTNB reagent (5,5′-Dithio-bis-(2-nitrobenzoic acid), 99%, D218200, Sigma®), (DAB) (4-(dimethylamino)benzaldehyde, ≥98%, 8.03057, Sigma®), ANS (8-Anilino-1-naphthalenesulfonic acid, ≥97%, A1028, Sigma®), SDS (Sodium dodecyl sulfate, ≥98.5%, L3771, Sigma®), 2,2′-Bipyridyl (99%, 798533, Sigma®), ferric solution (ammonium iron (III) sulfate dodecahydrate, 99%, 221260, Sigma®), sodium carbonate (P.A., Neon®), sodium salt hydrate of phytic acid (≥ 90% phosphorus, 68388, Sigma®), thioglycolic acid (P.A., Neon®), tannic acid (403040, Sigma®), vanillin (Nuclear®), L-Tryptophan (≥ 98% T0254, Sigma®), pronase® (protease from Streptomyces griseus, 4,000,000 PU/g, CAS no. 9036–06–0), trypsin (porcine pancreatic trypsin type IX-S, 13,000-20,000 BAEE units/mg protein, T0303, Sigma®), α-chymotrypsin (bovine pancreatic chymotrypsin type II, ≥ 40 units/mg protein, C4129, Sigma®), peptidase (porcine gastric mucosa pepsin, 3,200-4,500 units/mg protein, P6887, Sigma®), Criterion™ TGX™ Precast Midi Protein Gels (Bio-Rad Laboratories®), Laemmli sample buffer (2X, Bio-Rad®), β-mercaptoethanol (≥99%, Bio-Rad®), Bio-Safe™ Coomassie G-250 Stain (Bio-Rad®), Tris/Glycine/SDS electrophoresis buffer (10X, Bio-Rad®), and Precision Plus Protein™ Unstained Standards (Bio-Rad®).

# Figures and captions

Figure S1 – Zeta potential profile of Cowpea protein isolate across different pH values. The intersection with the dashed line at zero mV indicates the isoelectric point (IEP) of the protein matrix.


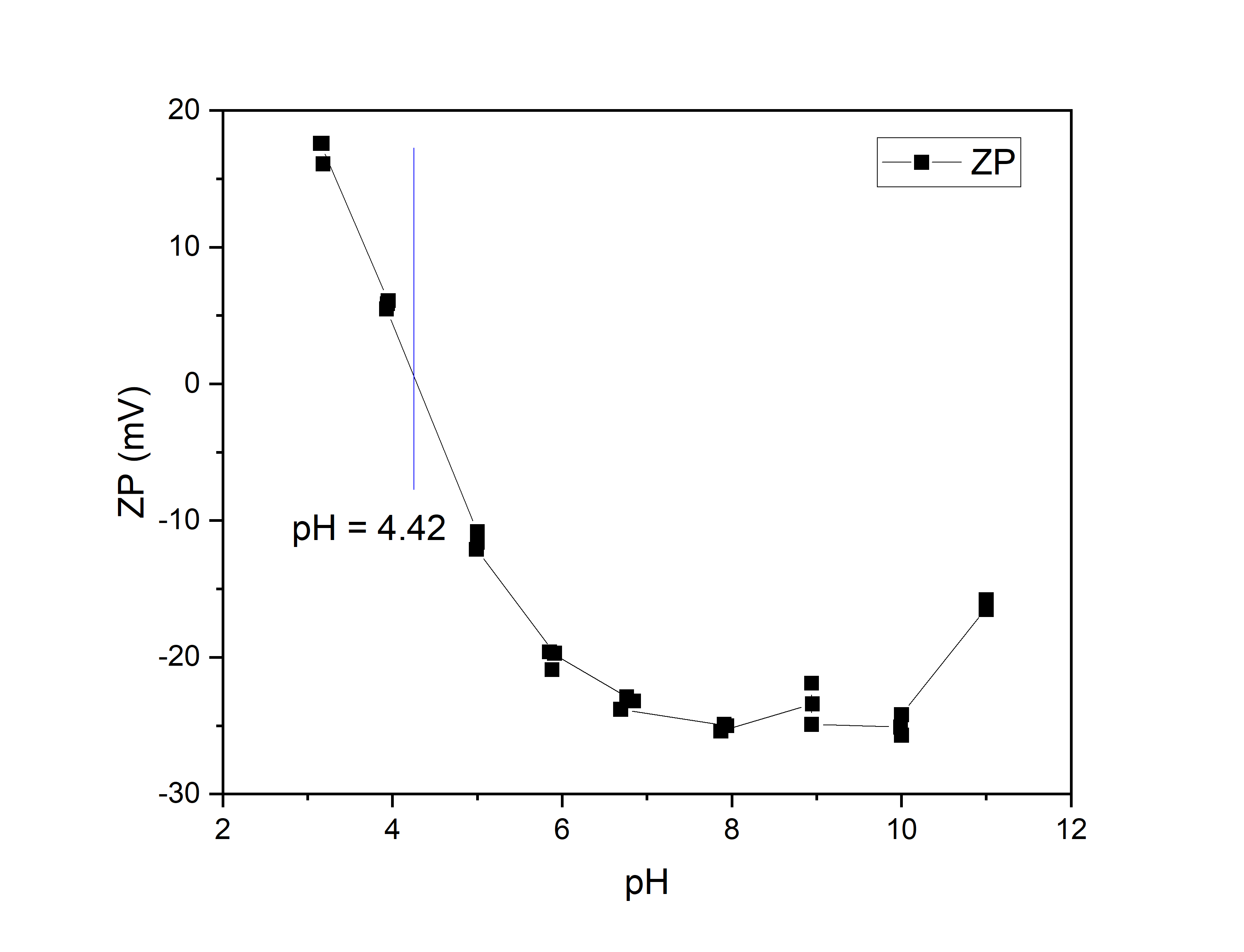


Figure S2 – Deconvoluted FTIR spectra of the Amide I region for cowpea protein concentrate (CPC), concentrate-soaked (CPC-S), and isolate (CPI). Shaded peaks represent the secondary structure motifs (random coil, β-turns, and β-sheets) obtained by Gaussian curve fitting.


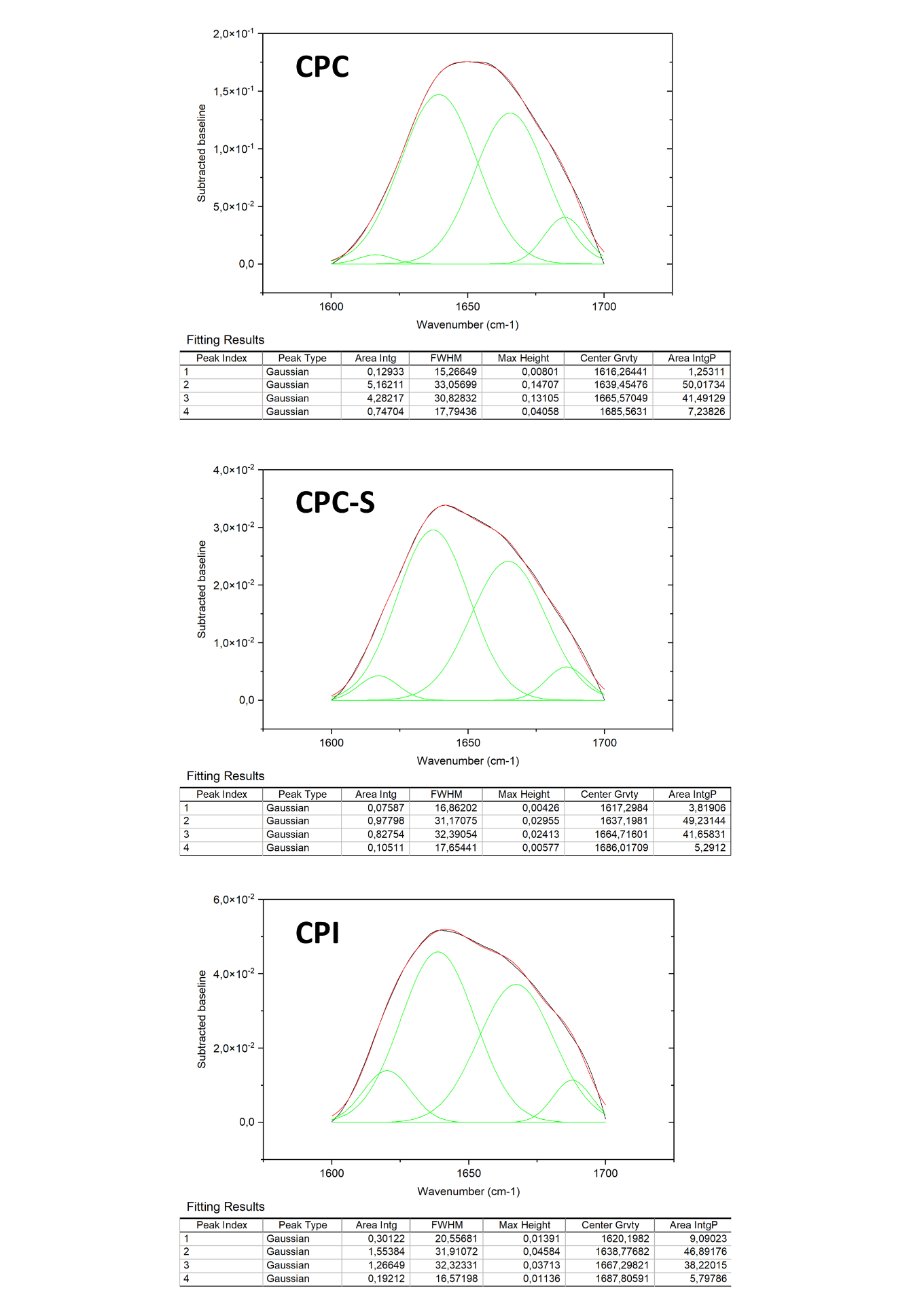


Figure S3 – DSC thermograms of cowpea protein ingredients.


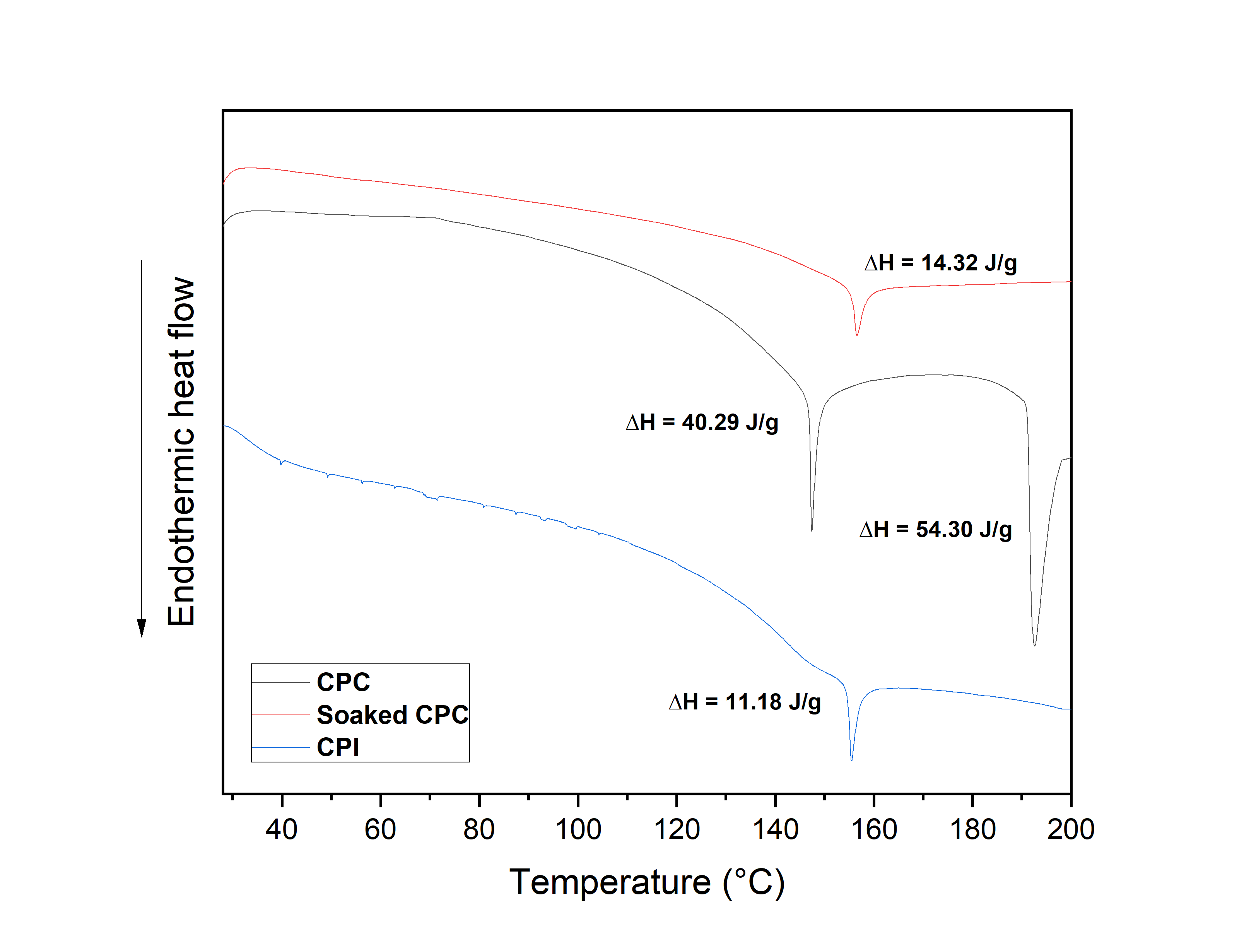


Note: CPC: cowpea protein concentrate; Soaked CPC: cowpea protein concentrate- soaked; CPI: cowpea protein isolate.
